# Supplementary material for: Splice-Junction-Based Mapping of Alternative Isoforms in the Human Proteome
Source: Cell Rep. Author manuscript; Available in PMC 2020 Jan 15. (PMC6961840; doi:10.1016/j.celrep.2019.11.026)
Supplement: 3 [file NIHMS1546469-supplement-3.zip › DF2/PXD0010154/AdrenalGland-36-Q15366-GVTIPYRPKPSSSPVIFAGGQAYTIQGQYAIPQPDLTK.pdf]

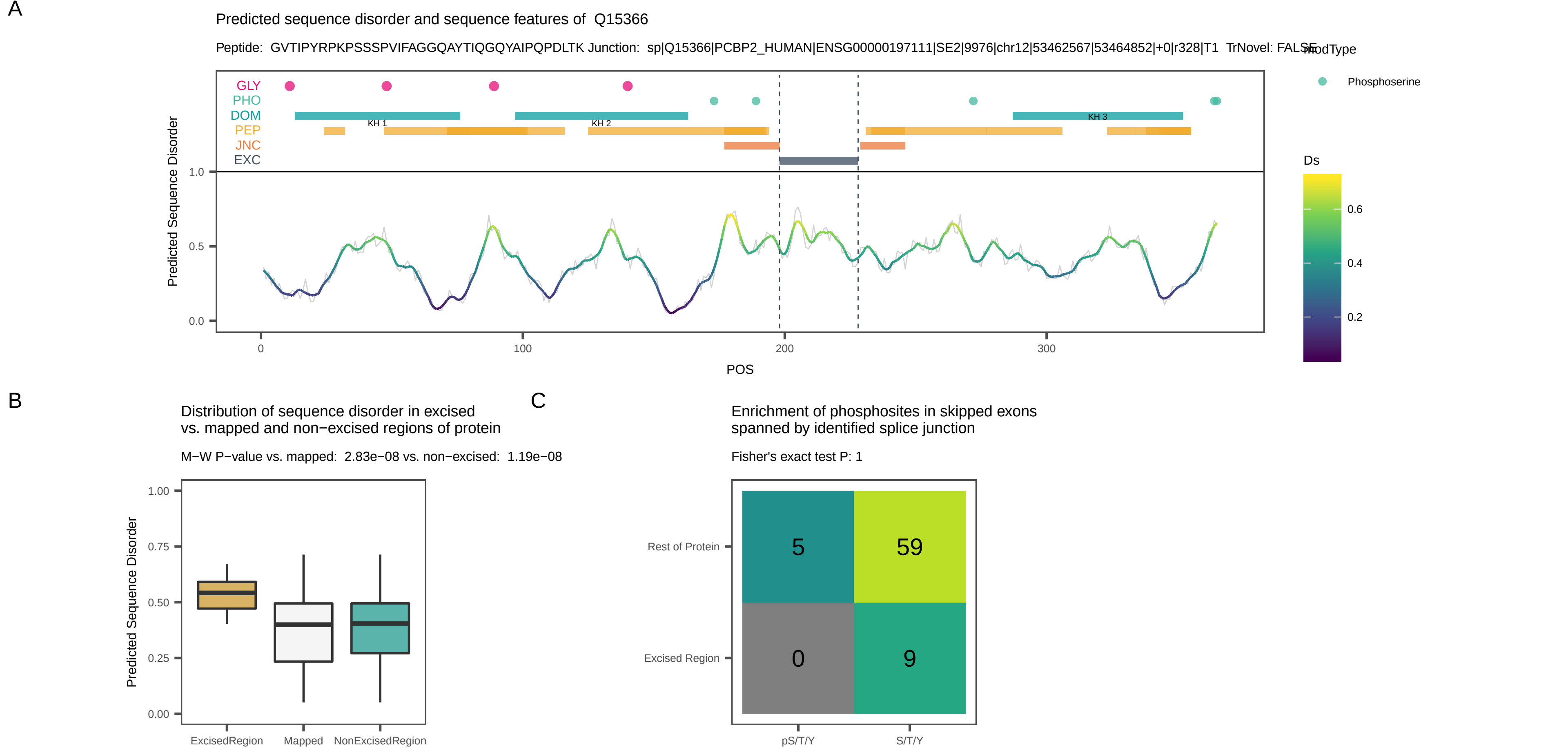
A
Predicted sequence disorder and sequence features of Q15366
Peptide: GVTIPYRPKPSSSPVIFAGGQAYTIQGQYAIPQPDLTK Junction: sp|Q15366|PCBP2_HUMAN|ENSG00000197111|SE2|9976|chr12|53462567|53464852|+0|r328|T1 TrNovel: FALSE
modType
Phosphoserine
GLY
PHO
DOM
PEP
JNC
EXC
KH 1
KH 2
KH 3
Predicted Sequence Disorder
1.0
0.5
0.0
0
100
200
300
POS
Ds
0.6
0.4
0.2
B
Distribution of sequence disorder in excised vs. mapped and non−excised regions of protein
M−W P−value vs. mapped: 2.83e−08 vs. non−excised: 1.19e−08
Predicted Sequence Disorder
1.00
0.75
0.50
0.25
0.00
ExcisedRegion
Mapped
NonExcisedRegion
C
Enrichment of phosphosites in skipped exons spanned by identified splice junction
Fisher's exact test P: 1
Rest of Protein
Excised Region
5
59
0
9
pS/T/Y
S/T/Y
